# Assessing the Dietary Habits of Canadians by Eating Location and Occasion: Findings from the Canadian Community Health Survey, Cycle 2.2

**DOI:** 10.3390/nu10060682

**Published:** 2018-05-27

**Authors:** Stephanie K. Nishi, Mahsa Jessri, Mary L’Abbé

**Affiliations:** 1 Clinical Nutrition & Risk Factor Modification Centre, St. Michael’s Hospital, Toronto, ON M5S 3E2, Canada; s.nishi@mail.utoronto.ca; 2 Department of Nutritional Sciences, Faculty of Medicine, University of Toronto, 150 College Street., Toronto, ON M5S 3E2, Canada; m.jessri@mail.utoronto.ca

**Keywords:** diet, food and nutrition, energy intake, dietary sodium, added sugars, dietary fat, food environment, Canadian

## Abstract

Occasion and location of food environment has an influence on dietary habits, nutritional quality and overall health and nutrition-related chronic disease risk. Eating occasion and location was assessed in 20,402 Canadians aged ≥ 2 years, with a focus on energy, saturated fat, added sugars, and sodium intake by age group. Data showed >80% of children, compared to ~60% of adolescents and adults, consumed three meals (breakfast, lunch, dinner) plus snacks in a day. Dinner contributed the most calories [ranging from 395 ± 11 kcal (2–3 year olds) to 952 ± 27 kcal (men 19–30 years)], saturated fat [7.4 ± 0.2% energy (2–3 year olds) to 9.1 ± 0.3% energy (women 31–50 years)], and sodium [851 ± 24 mg (2–3 year olds) to 1299 ± 69 mg (men 19–30 years)], while snacks contributed the most added sugars [22 ± 1 kcal (men >70 years) to 45 ± 1 kcal (2–3 year olds)]. By eating location, most Canadians (>90%) reported consuming food from home. Subsequently, home was associated with the majority of energy [1383 ± 23 kcal (women >70 years) to 2090 ± 35 kcal (boys 9–13 years)], saturated fat [20.4 ± 0.4%E (men 51–70 years) to 24.2 ± 0.4%E (2–3 year olds)], added sugars [77 ± 3 kcal (men 19–30 years) to 117 ± 2 kcal (2–3 year olds)], and sodium [2137 ± 59 mg (women 19–30 years) to 2638 ± 45 mg (men 51–70 years)] intakes. Reported eating behaviours suggest action is needed at individual and population levels to alter food purchasing and consumption habits, specifically with regards to snacking habits and foods prepared at home.

## 1. Introduction

Food environment has been declared a priority area for investigation and intervention by national and international organizations due their importance in influencing dietary habits and nutritional quality, as healthy eating is central to overall health and nutrition-related chronic disease risk reduction [1,2]. Food environments are the collective physical, economic, policy and socio-cultural surroundings, opportunities, and conditions that influence food choices and nutritional status [3]. At the macro-level, societal and cultural norms influence our eating occasions, meal patterns, and overall timing of food consumption. Generally, eating occasions can be described socially/culturally as breakfast, lunch, and dinner which represent “main meals”, while a smaller-sized meal is often considered a “snack”. At the physical environmental level, our eating patterns can be influenced by location of food preparation and consumption [4]. 

Studies have investigated the implications and patterns of various eating occasions, such as skipping breakfast or “snacking”; and eating locations, including consumption of fast foods and foods away-from-home. These dietary patterns in particular have been of interest since skipping breakfast, snacking, and consumption of fast foods and food from away-from-home locations have each been associated with lower diet quality and obesity among adults [5,6].

With the advent of a subsequent nutrition-focused Canadian Community Health Survey, having knowledge from the first such survey will provide the opportunity for future exploration of changes associated with the quality of foods consumed by Canadians, both from the individual food and nutrient level (as has been published [7,8,9,10]) and dietary habits, such as occasion and location of food consumption. Hence, the aim of this comprehensive analysis, is to assess the food choices and associated nutrient intakes (with a focus on sodium, fat, added sugar, and total energy intake due to their public health concern [11]) of Canadians according to meal occasion and location across the lifespan (e.g., children, adolescents, and adults), using the Canadian national nutrition survey (Canadian Community Health Survey, Cycle 2.2).

## 2. Materials and Methods

The CCHS 2.2 includes cross-sectional nutrition and health data for a nationally representative sample of 35,107 Canadians from all ten provinces. Participants were included in the current analysis if they were aged ≥2 years, not pregnant or breastfeeding, and had valid/complete dietary recalls based on Statistic Canada’s definition, where invalid/missing recalls are described as having extreme portion sizes and nutrient amounts or having incomplete meals and interviews. A modified version of the 5-step United States Department of Agriculture (USDA) Automated Multiple Pass Method was used to obtain detail 24-hour recall data. 24-hour dietary recall data were obtained using a modified version of the five-step US Department of Agriculture (USDA) Automated Multiple Pass Method (AMPM). Full details have been previously published [7,8,9,10,12]. For foods reported as mixed dishes, efforts were made to address this in the CCHS 2.2 via utilization of the recipe database to reflect the Canadian food supply and Canadian recipes. Since added sugars are not included in the CNF, the estimation method proposed by Brisbois et al. was used to derive estimates of added sugars [13]. This method uses CCHS data of total sugars and energy intakes to estimate added sugar intakes by categorizing the sugar content of food groups based on the source of the majority of sugars it contains as being either added or naturally occurring. For instance, it assumed that all sugars in fruits, vegetables, milk, and 100% fruit juice are naturally occurring, whereas all sugars in confectionary, sugars, fruit drinks and cereals/grains are considered added. Using published International Glycemic Index (GI) tables, the GI values of reported foods were calculated [14] and assigned to each of the Bureau of Nutritional Sciences (BNS) food categories [15] using the procedures suggested by Louie et al. and Flood et al. [16,17]. Glycemic load was calculated by multiplying the glycemic index value by the number of grams of carbohydrate then dividing by 100 [14,18]. Energy adjustments were performed to reduce the probability of observing results influenced and derived merely by calorie intakes. Physical activity assessment has been previously reported [7,8].

Trained interviewers measured height and weight according to standard protocols, and body mass index (BMI) was calculated dividing subjects’ weight by height squared [19]. Descriptive analyses were stratified by sex and age categories, as defined in the IOM Dietary Reference Intakes (DRI) [20].

Occasion and location of eating were defined based on the Statistics Canada definitions and new definitions proposed by Leech et al. [21,22]. CCHS respondents were asked where the food they ate had been prepared: home, which includes an individual’s home and someone else’s home; fast food, which includes fast food restaurants, take-out; and other locations. Other locations include: restaurants with waiter/waitress; other restaurants; bars, taverns, lounges; school and non-school cafeterias; work; child care centres; family/adult care centres; vending machines; grocery, corner, or other types of stores. Occasion was defined by CCHS respondents and included three main meals (breakfast, lunch, dinner) and snacks.

When discussing “consumers only” this refers to the fact that the population included in the particular analysis being discussed were only those that ate at the specified location or occasion being examined. Conversely, non-consumers represent individuals that did not obtain any of their daily kilocalories at the specified location or occasion.

To identify potential implausible dietary reporting, each participant in this study was categorized as an under-reporter, plausible reporter or over-reporter based on the comparison of their total estimated energy requirement (EER) and their reported energy intake (EI) [20], as has been described in detail previously [7,8].

Nutritional quality was evaluated using Health Canada’s Surveillance Tool Tier System [23], as described previously [7,8].

Statistical Analysis System (SAS) software (version 9.4; SAS Institute Inc., Cary, NC, USA) was used to perform all statistical analyses. All analyses were weighted to obtain estimates at a population level. Survey weights were calculated by Statistics Canada based on respondent classes with similar socio-demographic characteristics to maintain a nationally representative sample. The bootstrap balanced repeated replication (BRR) technique was used, as recommended by Statistics Canada, to account for the complex survey design [24]. To assess the lifestyle and dietary characteristic of participants, PROC SURVEYREG and PROC SURVEYLOGISTIC were used to analyse continuous and categorical variables, respectively. Group comparison with Tukey post-hoc adjustment was used to evaluate the characteristics of participants classified within DRI age and sex categories.

## 3. Results

This study included 4215 children (aged >2 to <12 years), 4649 adolescents (aged >12 to <19 years), and 11,538 adults (aged ≥19 years) for a total of 20,402 Canadians aged >2 years. 

### 3.1. Eating Occasion

**Figure 1:**
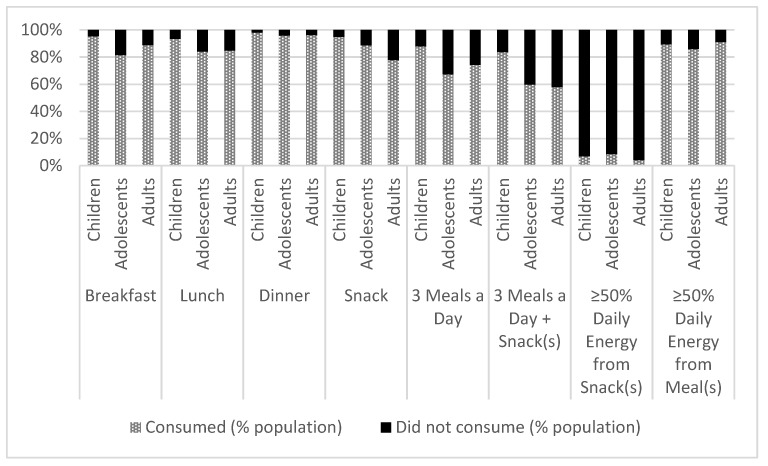
Percentage of meal consumption at different occasions, by age group, adjusted for sex (*n* = 20,402).

Figure 1 shows the percentage of the population who consumed versus did not consume food and/or beverage(s) at a particular eating occasion by age group. In general, at least 80% of the population reported eating something at each of the main eating occasions (i.e., breakfast, lunch, dinner), or as a snack during the day. Adolescents and adults were more likely than children to not consume the 3 main meals (breakfast, lunch, and dinner) in a day, skipping breakfast or lunch more often than children. Nearly 60% of adolescents and adults and over 80% of children consumed 3 meals a day plus snack(s), although nearly 10% of this representative sample of the Canadian population consumed more than 50% of their calories from snacks. Across all age groups, those who skipped breakfast and/or consumed over 50% of their daily energy intake during snack occasions were more likely to underreport their daily energy consumption. Adolescents and adults who skipped breakfast were more likely to participate in less physical activity (i.e., the percentage of adolescents and adults that did not consume breakfast and participated in physical activity ≤1 day/week was: adolescents 42 ± 3%, adults 64 ± 2%, *p* < 0.05, respectively) and more likely to be a daily smoker (adolescents 6.3 ± 1.2%, adults 31.5 ± 2.6%, *p* < 0.005) compared with breakfast consumers.

Across all age groups, total energy consumption by occasion showed dinner contributed the highest amount of daily calories consumed (children: 29%, adolescents: 33%, adults: 36%), followed by lunch (children: 25%, adolescents: 23%, adults: 24%), snacks (children: 25%, adolescents: 23%, adults: 16%), and breakfast (children: 18%, adolescents: 17%, adults: 18%). Energy from snacking was comparable to the amount of energy obtained from ‘main meals’ (breakfast, lunch, dinner), and was highest among young children and adolescents and contributed to 27% of total energy among boys and girls 2–3 years, while it only contributed to 13% of energy among males over the age of 70 years.

Similar to energy, the distribution of saturated fat intake from snacking was comparable to that consumed at main meals. Saturated fat intake at breakfast ranged from 3.2 g ± 0.1 g (women, >70 years) to 5.3 g ± 0.3 g (in boys, 14–18 years, and men, 31–50 years), while lunch [4.8 g ± 0.2 g (children, 2–3 years) to 8.0 g ± 0.3 g (boys, 9–13 years)], dinner [5.2 g ± 0.2 g (children, 2–3 years) to 11.8 g ± 0.4 g (boys, 14–18 years)], and snack(s) [2.8 g ± 0.2 g (women, >70 years) to 9.4 g ± 0.4 g (boys, 14–18 years)] tended to be associated with providing more of the daily saturated fat intake (Table 1).

Snack based added sugar consumption was higher than added sugar consumption at each of the main meals (breakfast, lunch, dinner) across all DRI age and sex-based groups except for in individuals over 70 years of age, which was particularly pronounced in the <19-year-olds (Table 1). Among those under 18 years of age, ~~8-10% of total energy intakes as added sugars were consumed as snacks compared to ~4-7% in those over 18 years of age. At breakfast, added sugar consumption ranged from about 20–30% for all age groups.

Sodium consumption by occasion and DRI age and sex-based group, is presented in Table 1. Similar to energy, dinner contributed the most daily sodium (Range 395 ± 11 to 951 ± 27 mg), followed by lunch (361 ± 9 to 652 ± 18 mg), snacks (207 ± 8 to 718 ± 25 mg) and breakfast (294 ± 7 to 458 ± 14 mg). Of the most frequently consumed types of foods by eating occasion, “other foods” were within the top 3 types of foods consumed at all eating occasions except for breakfast. “Other foods” comprise of foods and beverages not considered part of the four main food groups of Canada’s Food Guide, including saturated and/or trans fats and oils (e.g., butter), beverages (e.g., carbonated drinks), uncategorized foods (e.g., ingredients, seasonings and unprepared foods), alcoholic beverages, and high fat and/or high sugar foods (e.g., candies).

Those that consumed majority of their energy consumption during snack occasions compared to main meals had significant differences in nutrient intakes compared to those who obtained the majority of their calories during main meal (i.e., breakfast, lunch, dinner) eating occasions (Table 2).

Body mass index (BMI) was not significantly different between those who skipped breakfast compared to those who consumed breakfast (*p* > 0.05). However, BMI was significantly lower in children and adolescents who consumed greater than 50% of their daily energy as snacks compared to those who did not consume 50% or more of their daily energy intake at snack occasions when adjusted for age, sex, dietary reporting status, and energy intake (*p* < 0.05).

### 3.2. Eating Location

**Figure 2:**
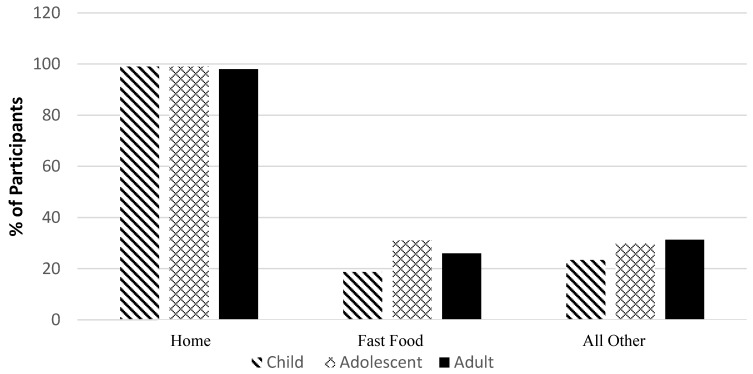
Prevalence of eating at different locations* by age group, adjusted for sex. (*n* = 20,402). *Location refers to the place of food preparation, which is not necessarily the same location as consumption. For instance, food could have been prepared at home, yet consumed at work and since it was consumed at work, the location of consumption would be noted as “all other”. “Home” refers to foods prepared at the participant’s home or at someone else’s home. “Fast Food” refers to foods prepared at fast food/pizza restaurants and take-out. “All other” locations refer to the following locations: restaurants with waiter/waitress; bar/tavern/lounge; vending machine; restaurants with no additional information; cafeteria not at school; cafeteria at school; child care centre; family/adult care centre; other; grocery, corner or other types of stores; or at work. Children = aged >2 to <12 years, adolescents = aged >12 to <19 years, adults = aged >19 years.

Figure 2 shows the percentage of the population who consumed at least one food or beverage item at the location noted during the day recorded, for each of the locations assessed (home, fast food, or other). Over 90% of participants consumed a food or beverage that had been prepared at home, although on any given day, 20–30% of the population also consumed food prepared at a fast food location as did a similar proportion at other locations. Between 40–60% of the population consumed at least something outside the home on any given day. There were no significant differences observed in terms of the physical activity levels among children, adolescents or adults in those who consumed the ≥50% of their daily calories intake from foods prepared at home compared to those who consumed <50% of their daily energy from home-prepared foods. This lack of significance in physical activity level was also seen when comparing those who consumed <50% daily energy as fast food and those that ate ≥50% of their daily energy as fast food across all age groups. Whereas, adults who ate ≥50% of their daily calories as fast food where more likely to have never smoked (47 ± 1%) and also comprised a lower percentage of daily smokers (21 ± 1%) compared to those that ate <50% of their daily energy as fast food (36 ± 4 % and 29 ± 4%, respectively) (*p* <0.05).

The majority of energy (kilocalorie) intake was consumed from foods prepared at home across all age groups (Table 3). In children and adolescents (boys and girls aged 2 to 18 years) 54 to 62% of total energy consumed was from foods prepared at home; compared to fast food locations where the percentage of daily energy consumed ranged from 16–26% of total calories; while the daily energy consumption from food prepared at other locations ranged 18–23% of total calories, for those who ate foods at these locations. Very similar trends were observed in adults (≥19 years). Among consumers who ate foods prepared outside the home, young males 19–30 years consumed the greatest amount of calories (nearly 50%) from foods prepared at locations other than at home. 

When eating at fast food locations, boys and girls aged 2–3 years consumed on average a lower percentage of energy as SFA (17–20%), whereas 14–18-year-old boys consumed the most at 28% of total SFA. Consumption of foods prepared at other locations contributed approximately 7–12% of total energy intakes as SFAs. 

The majority of Canadians consumed more than the recommended 10% of total daily calories from saturated fat. The only DRI based age and sex groups which did not on average consume more than the recommended 10% of total daily calories from saturated fat were: Men 19–30 years; Women 51–70 years; and Men >70 years.

In all age groups, most added sugar in a day was consumed from foods prepared at home, compared to fast food and other locations (Table 3). From foods prepared at home, added sugar ranged from 48% of total added sugar intake among men 19–30 years to 63% of total consumption by 2–3-year-olds, men 51–70 years, and women ≥70 years. Compared to fast food and other locations where children 2 to 3 years consumed 15% and 21% of their total added sugar intakes, respectively. Among foods prepared at other locations, boys 9–13 years consumed the highest amount of percentage added sugar intake (25%) compared to other DRI groups. Fast food locations were the place of 14–28% of total added sugar intakes, where men ≥70 years had the lowest percentage intake (14%) and boys 14–18 years and 19–30-year-old men had the highest amount at fast food locations (28%). Overall, the mean amount of added sugars based on DRI age and sex groups are presented in Table 3. In general, these mean intakes are <10% for adults ≥31 years, except for women ≥70 years, and exceed 10% for all children, adolescents, and adults ≤30 years.

Across all DRI age and sex-based groups, >70% of sodium consumed was associated with foods prepared at home, ranging from to 1731 mg ± 40 mg in children, 2–3 years, to 2896 mg ± 108 mg in men 19 to 30 years (Table 4). Men 19–30 years also had the highest amount of sodium consumed from foods prepared at fast food restaurants (438 mg ± 35 mg) and take-out locations (37.6 mg ± 11.5 mg). In terms of milligrams, in a day, sodium intake on average met or exceeded 2000 mg at home alone in all age groups except children 2–3 years. Health Canada recommends people over the age of one year consume between 1000–1500 mg of sodium per day to obtain an “Adequate Intake” (AI) of sodium, while people aged 14 and over should not consume more than the “Tolerable Upper Intake Level” (UL) of 2300 mg of sodium per day. Overall, the average sodium intake per day by all DRI age and sex groups surpassed their recommended tolerable upper intake level [25].

When the 10 most commonly consumed types of foods were assessed by location of food preparation (home, fast food, and other locations) in children, adolescents, and adults “other foods” were the most prevalent from all locations assessed.

Eating mainly foods prepared at home or at fast food locations were not associated with overall BMI status of the population assessed, in both unadjusted and adjusted (age, sex, dietary reporting status, and energy) models (*p* > 0.05) (data not shown).

## 4. Discussion

This study presents findings from a nationally representative sample of Canadians from the CCHS, Cycle 2.2 of nutrient intakes by food consumption location and occasion according to DRI-based age and sex classifications. 

According to eating occasion, our findings suggest breakfast skipping was not associated with being overweight or obese, thus differing from the literature regarding this dietary behaviour and overweight and obesity risk [26,27]. Unexpectedly, the results also showed that BMI was significantly lower in children and adolescents who consumed greater than 50% of their daily energy as snacks compared to those who did not, although the difference was not clinically significant. Several studies have shown that frequent snacking is associated with higher total caloric intake [28,29]; however, similar to the findings of this study, not all studies indicate a relation between snacking behaviour and increased weight status [30]. A potential reason for this is that frequent snacking may have little impact on overall daily energy intake or weight status for those who may consume snacks in place of meals, or if healthy foods, such as fruit, are frequently consumed as snacks. 

In respect to eating location, findings indicated that despite obtaining the majority of food either prepared and/or consumed at home, the nutritional quality of foods consumed was not meeting recommendations for saturated fat, added sugars, or sodium intake across the majority of age and sex groups despite the common thought that consuming home-prepared foods is “healthier”. Studies have shown that increasingly unhealthy home cooking norms may be a contributing factor along with ready-made meals and packaged, processed food items that can be easily prepared or assembled for consumption at home [31,32]. As well, while the majority of sodium was consumed “at home” this does not say that restaurant foods have low sodium, but rather it shows that on a population basis, Canadians are preparing the majority of their foods at home and hence consuming the majority of their sodium intake from home locations compared to restaurants, and other locations, however, the combined sodium intakes from foods consumed away from home for some age groups nears that from at home, e.g., teenage boys and young men.

A major strength of this study is that it represents a comprehensive analysis of nutrient intakes according to meal occasion and eating location and some dietary patterns, such as skipping breakfast, of Canadians by DRI-based age and sex group in a large nationally-representative sample. Including several covariates, measured anthropometry, and adjusting for misreporting [10].

Weaknesses of this study include the fact that the CCHS data was collected in 2004/2005, over a decade prior to these analyses, although it will provide a detailed assessment of eating patterns on which to monitor trends compared to the CCHS 2015 data. There is also the limitation of using a single day dietary report which does not reflect usual intakes and are memory dependent, which may lead to under- or over-reporting; however, a single 24-hour recall is sufficient to report mean group intake [33]. Additionally, when responding to the dietary consumption question, some respondents may have provided information about the location where they consumed the food rather than the place where it had been prepared. If a respondent reported having eaten at home, he or she may have actually purchased the food elsewhere and brought it home to consume and reported the food as “prepared at home”.

## 5. Conclusions

The observed eating behaviours and patterns of Canadians suggest policy and educational action is needed at the individual and population levels to alter food purchasing and consumption habits, specifically in regards to snacking habits and foods prepared at home. Such strategies would help Canadians to more easily make food choices in line with the recommendations for healthy eating promoted by Health Canada. While this data is cross-sectional, and longitudinal studies are needed to further elucidate the role food environment, including eating occasion and location, have on dietary habits and chronic disease risk of Canadians, with the release of the CCHS 2015 data, the present analyses and findings provide a foundation for evaluating dietary trends in Canada.

## Figures and Tables

**Table 1:** Dietary intake recall by Dietary Reference Intake (DRI) age and sex group by eating occasion.

	Energy (kcal)				Saturated Fat (g)				Added Sugars (g)				Sodium (mg)			
DRI	Breakfast	Lunch	Dinner	Snack	Breakfast	Lunch	Dinner	Snack	Breakfast	Lunch	Dinner	Snack	Breakfast	Lunch	Dinner	Snack
Boys & Girls, 2–3 year	294 (7)	361 (9)	395 (11)	391 (15)	3.7 (0.2)	4.8 (0.2)	5.2 (0.2)	5.2 (0.3)	23.1 (0.8)	17.6 (0.9)	16.3 (0.7)	36.9 (1.5)	357 (11)	641 (24)	623 (23)	340 (15)
Boys & Girls, 4–8 year	337 (5)	486 (8)	558 (11)	454 (10)	3.8 (0.1)	6.5 (0.2)	7.3 (0.2)	5.9 (0.2)	25.8 (0.5)	26.4 (0.8)	22.9 (0.6)	39.3 (1.0)	424 (8)	862 (19)	911 (31)	435 (14)
Boys, 9–13 year	420 (11)	608 (19)	755 (17)	565 (18)	4.6 (0.2)	8.0 (0.3)	9.6 (0.3)	7.1 (0.3)	32.7 (1.3)	30.7 (1.3)	30.6 (1.1)	46.8 (1.7)	541 (18)	1049 (31)	1232 (34)	572 (26)
Girls, 9–13 year	336 (9)	503 (12)	650 (16)	482 (15)	3.7 (0.2)	6.3 (0.2)	8.3 (0.3)	6.1 (0.3)	24.5 (0.9)	26.8 (0.9)	26.7 (0.9)	42.1 (1.7)	442 (14)	911 (34)	1976 (39)	471 (20)
Boys, 14–18 year	458 (14)	653 (18)	910 (18)	718 (25)	5.3 (0.3)	8.0 (0.3)	11.8 (0.4)	9.4 (0.4)	32.5 (1.3)	33.5 (1.2)	35.3 (1.3)	52.3 (2.3)	616 (24)	1123 (36)	1476 (41)	814 (39)
Girls, 14–18 year	321 (10)	475 (12)	668 (13)	486 (18)	3.5 (0.2)	5.7 (0.2)	7.8 (0.2)	6.5 (0.4)	22.9 (1.0)	24.2 (0.9)	27.2 (1.1)	39.2 (1.7)	398 (16)	866 (31)	1104 (30)	500 (23)
Men, 19–30 year	430 (16)	605 (21)	952 (27)	515 (23)	5.2 (0.3)	7.0 (0.3)	11.3 (0.5)	6.4 (0.3)	26.4 (1.2)	26.7 (1.4)	30.3 (1.1)	34.2 (1.8)	602 (33)	1136 (63)	1624 (74)	584 (32)
Women, 19–30 year	316 (12)	445 (17)	628 (16)	379 (16)	3.7 (0.3)	5.3 (0.3)	7.8 (0.3)	4.7 (0.3)	22.2 (1.1)	19.4 (0.9)	22.4 (0.9)	28.6 (1.4)	399 (20)	852 (53)	1009 (29)	384 (23)
Men, 31–50 year	414 (12)	622 (18)	886 (21)	409 (18)	5.3 (0.3)	7.9 (0.3)	10.6 (0.4)	5.2 (0.3)	23.3 (1.0)	24.6 (1.1)	25.7 (1.0)	30.0 (1.6)	572 (24)	1117 (38)	1407 (45)	423 (26)
Women, 31–50 year	303 (8)	417 (12)	684 (18)	340 (13)	3.8 (0.2)	5.0 (0.2)	8.2 (0.3)	4.7 (0.3)	17.5 (0.8)	17.6 (0.9)	20.8 (0.9)	25.2 (1.1)	403 (14)	810 (27)	1165 (34)	327 (17)
Men, 51–70 year	371 (9)	504 (14)	823 (19)	358 (14)	4.4 (0.2)	6.4 (0.3)	9.7 (0.3)	4.7 (0.3)	21.3 (0.7)	20.3 (0.9)	25.0 (1.0)	25.0 (1.2)	518 (15)	1060 (39)	1318 (38)	364 (18)
Women, 51–70 year	311 (8)	416 (11)	620 (12)	264 (9)	3.3 (0.1)	5.2 (0.2)	7.3 (0.2)	3.2 (0.2)	18.8 (0.6)	17.0 (0.6)	20.5 (0.7)	21.2 (0.8)	404 (11)	799 (31)	1067 (26)	237 (12)
Men, >70 year	406 (10)	483 (16)	676 (20)	237 (12)	4.5 (0.2)	5.9 (0.3)	8.1 (0.4)	3.1 (0.2)	25.8 (1.2)	18.5 (0.9)	25.6 (1.2)	18.8 (1.1)	521 (18)	962 (35)	1138 (42)	204 (15)
Women, >70 year	318 (7)	416 (11)	526 (11)	208 (8)	3.2 (0.1)	5.3 (0.3)	6.1 (0.2)	2.8 (0.2)	21.4 (0.7)	17.3 (0.7)	21.4 (0.7)	17.0 (0.7)	406 (15)	807 (33)	878 (26)	176 (8)

Data are presented as Mean (Standard Error of the Mean). Eating occasion definitions are based on those described in the CCHS 2.2 Nutrition—General Health and 24-Hour Dietary Recall: Data Dictionary. Results presented are on a per capita (consumers only) basis to avoid the impact of non-consumers (zero-inflation) on the total model and to capture the eating behaviours of those who presented with different eating behaviours, as was done for the US Dietary Guidelines [19].

**Table 2:** Dietary intakes of Canadians who consumed ≥50% of daily kcal during snack occasions compared to those who did not, by age group.

		Children (≥2 to <12 years, *n* = 4215)			Adolescents (≥12 to <19 years, *n* = 4649)			Adults (aged ≥ 19 years, *n* = 11538)		
Variable	Units	<50% kcal/d from snacks *	≥50% kcal/d from snacks ^†^	Pr > F	<50% kcal/d from snacks *	≥50% kcal/d from snacks ^†^	Pr > F	<50% kcal/d from snacks *	≥50% kcal/d from snacks ^†^	Pr > F
Energy	Kcal	1771 (12)	1935 (55)	0.005	2490 (24)	2741 (95)	0.008	2418 (19)	2488 (64)	0.258
Protein	%Energy	16.0 (0.1)	12.8 (0.3)	<0.0001	14.4 (0.1)	11.7 (0.4)	<0.0001	14.8 (0.1)	12.6 (0.4)	<0.0001
Fat	%Energy	31.3 (0.2)	33.6 (0.6)	0.000	29.7 (0.2)	28.2 (0.8)	0.048	31.9 (0.2)	35.2 (0.9)	0.000
Saturated Fat	%Energy	10.9 (0.1)	9.6 (0.4)	0.004	10.5 (0.1)	10.7 (0.4)	0.677	10.3 (0.1)	10.8 (0.4)	0.237
Carbohydrate	%Energy	55.9 (0.3)	60.1 (0.9)	<0.0001	53.7 (0.3)	55.5 (1.0)	0.059	48.5 (0.3)	50.0 (0.9)	0.122
MUFA	%Energy	11.3 (0.1)	11.0 (0.4)	0.418	12.2 (0.1)	12.4 (0.3)	0.465	12.7 (0.1)	14.6 (0.5)	0.001
PUFA	%Energy	4.6 (0.1)	4.9 (0.2)	0.183	5.2 (0.1)	5.7 (0.3)	0.054	5.7 (0.1)	6.7 (0.3)	0.000
Fiber	g/1000 kcal	7.4 (0.1)	7.7 (0.4)	0.290	6.7 (0.1)	6.8 (0.2)	0.677	8.9 (0.1)	8.1 (0.4)	0.023
Alcohol	%Energy	0.0 (0.0)	0.0 (0.0)	0.258	0.6 (0.1)	0.1 (0.1)	0.032	3.0 (0.2)	1.8 (0.6)	0.060
Vitamin A	RE/1000 kcal	319 (6)	266 (14)	0.000	280 (6)	246 (24)	0.181	370 (10)	298 (16)	<0.0001
Vitamin D	ug/1000 kcal	3.3 (0.1)	2.4 (0.2)	<0.0001	2.7 (0.1)	2.0 (0.1)	<0.0001	3.0 (0.1)	2.2 (0.2)	<0.0001
Thiamin	mg/1000 kcal	0.9 (0.0)	0.7 (0.0)	<0.0001	0.9 (0.0)	0.8 (0.0)	0.000	0.8 (0.0)	0.7 (0.0)	<0.0001
Riboflavin	mg/1000 kcal	1.1 (0.0)	0.9 (0.0)	<0.0001	1.0 (0.0)	0.8 (0.0)	<0.0001	1.0 (0.0)	0.9 (0.0)	0.003
Niacin	NE/1000 kcal	15.9 (0.2)	13.7 (0.4)	<0.0001	16.6 (0.2)	14.1 (0.5)	<0.0001	19.8 (0.2)	16.0 (0.5)	<0.0001
Vitamin B6	mg/1000 kcal	0.8 (0.0)	0.8 (0.0)	0.155	0.8 (0.0)	0.6 (0.0)	<0.0001	1.0 (0.0)	0.8 (0.0)	<0.0001
Folate	ug/1000 kcal	94.7 (2.1)	90.5 (6.8)	0.529	90.7 (1.6)	82.3 (4.3)	0.047	120 (2)	100 (5)	<0.0001
Vitamin B12	ug/1000 kcal	1.9 (0.0)	1.5 (0.2)	0.053	1.8 (0.0)	1.4 (0.1)	<0.0001	2.2 (0.1)	1.4 (0.1)	<0.0001
Vitamin C	mg/1000 kcal	77.7 (2.8)	77.6 (7.7)	0.990	67.4 (2.1)	54.9 (4.1)	0.006	63.1 (1.1)	58.1 (6.3)	0.446
Calcium	mg/1000 kcal	552 (8)	446 (19)	<0.0001	473 (7)	413 (16)	0.000	416 (4)	373 (17)	0.009
Phosphorus	mg/1000 kcal	669 (7)	591 (17)	<0.0001	619 (6)	565 (15)	0.001	664 (5)	575 (16)	<0.0001
Potassium	mg/1000 kcal	1404 (15)	1305 (54)	0.047	1308 (14)	1164 (37)	0.000	1583 (12)	1359 (36)	<0.0001
Sodium	mg/1000 kcal	1434 (18)	1319 (63)	0.064	1466 (14)	1365 (39)	0.014	1544 (18)	1405 (45)	0.003
Magnesium	mg/1000 kcal	138 (2)	133 (4)	0.070	131 (1)	124 (3)	0.039	167 (1)	153 (5)	0.004
Iron	mg/1000 kcal	6.9 (0.1)	6.5 (0.2)	0.182	6.8 (0.1)	6.0 (0.2)	<0.0001	7.0 (0.1)	6.2 (0.2)	0.000
Zinc	mg/1000 kcal	5.0 (0.1)	4.1 (0.2)	0.000	4.9 (0.1)	4.2 (0.1)	<0.0001	5.6 (0.1)	4.5 (0.1)	<0.0001
Added Sugar	%Energy	13.2 (0.4)	17.5 (1.3)	0.000	14.4 (0.3)	16.9 (1.1)	0.024	9.1 (0.2)	13.5 (0.9)	<0.0001
Glycemic Index	GI	54.7 (0.2)	55.7 (0.5)	0.064	55.8 (0.2)	56.1 (0.6)	0.655	52.6 (0.2)	53.4 (0.7)	0.248
Glycemic Load	GL	136 (1)	168 (7)	<0.0001	188 (2)	215 (8)	0.001	155 (2)	171 (7)	0.034
Energy Density	kcal/g	2.0 (0.0)	2.1 (0.1)	0.061	2.1 (0.0)	2.3 (0.1)	0.005	1.8 (0.0)	2.3 (0.1)	<0.0001

Data are presented as mean (SEM), and adjusted for age, sex, dietary reporting status, and energy where indicated by the variable units. * did not consume ≥50% of daily calories at “snack” occasions. ^†^ consumed ≥50% of daily calories at “snack” occasions.

**Table 3:** 24-hour dietary intake recall by Dietary Reference Intake (DRI) age and sex group by location of food preparation for total energy, saturated fat, and added sugar intake.

	Energy (kcal)			Saturated Fat (g)			Added Sugars (g)		
DRI	Home *	Fast Food ^†^	All Other ^‡^	Home *	Fast Food ^†^	All Other ^‡^	Home *	Fast Food ^†^	All Other ^‡^
Boys & Girls, 2–3 year	1411 (23)	377 (29)	505 (36)	19.0 (0.4)	5.0 (0.5)	7.1 (0.6)	98 (2)	21 (3)	31 (2)
Boys & Girls, 4–8 year	1689 (19)	529 (23)	502 (27)	21.5 (0.4)	7.0 (0.4)	6.7 (0.4)	109 (2)	33 (2)	33 (2)
Boys, 9–13 year	2090 (35)	761 (31)	812 (83)	25.7 (0.6)	10.4 (0.5)	11.3 (1.4)	130 (3)	46 (3)	59 (6)
Girls, 9–13 year	1786 (29)	617 (29)	588 (41)	21.8 (0.5)	7.6 (0.5)	8.7 (1.0)	113 (2)	37 (2)	41 (3)
Boys, 14–18 year	2307 (45)	1082 (38)	795 (40)	28.5 (0.7)	13.4 (0.6)	10.9 (0.8)	134 (3)	68 (4)	57 (3)
Girls, 14–18 year	1615 (28)	692 (26)	673 (42)	19.1 (0.5)	8.7 (0.4)	8.6 (0.7)	99 (2)	44 (3)	43 (4)
Men, 19–30 year	2038 (48)	958 (48)	1014 (57)	23.3 (0.8)	12.0 (0.7)	11.5 (0.7)	103 (3)	52 (3)	49 (5)
Women, 19–30 year	1498 (36)	651 (30)	598 (35)	17.5 (0.7)	8.7 (0.5)	6.9 (0.4)	87 (2)	33 (2)	32 (2)
Men, 31–50 year	2036 (38)	767 (43)	722 (44)	24.0 (0.7)	10.8 (0.7)	9.1 (0.6)	97 (3)	36 (2)	34 (2)
Women, 31–50 year	1509 (31)	498 (23)	720 (42)	18.3 (0.6)	7.4 (0.5)	8.5 (0.5)	78 (2)	24 (2)	30 (2)
Men, 51–70 year	1892 (31)	504 (32)	715 (41)	21.9 (0.6)	7.4 (0.6)	10.0 (0.8)	89 (2)	23 (2)	28 (2)
Women, 51–70 year	1464 (22)	484 (39)	580 (32)	16.8 (0.4)	7.4 (0.7)	6.8 (0.5)	76 (1)	18 (1)	27 (2)
Men, >70 year	1676 (35)	464 (37)	785 (105)	19.5 (0.7)	6.2 (0.7)	10.4 (1.7)	87 (2)	17 (2)	30 (8)
Women, >70 year	1383 (22)	370 (30)	608 (42)	16.1 (0.4)	4.8 (0.4)	7.7 (0.5)	77 (2)	16 (2)	26 (2)

Data are presented as Mean (Standard Error of the Mean). * ‘Home’ includes an individual’s home and someone else’s home; ^†^ ‘Fast Food’ includes fast food restaurants, take-out; and other locations. ^‡^ ‘All Other’ locations include: restaurants with waiter/waitress; other restaurants; bars, taverns, lounges; school and non-school cafeterias; work; child care centres; family/adult care centres; vending machines; grocery, corner, or other types of stores.

**Table 4:** 24-hour dietary intake recall by Dietary Reference Intake (DRI) age and sex group by location of food preparation for sodium intake.

	Sodium (mg)														
DRI	Home	Someone Else’s Home	Restaurant, Fast Food/Pizza	Take-Out	Restaurant with Waiter	Bar/Tavern/Lounge	Vending Machine	Restaurant No Additional Info	Cafeteria, Not At School	Cafeteria, at School	Child Care Centre	Family/Adult Care Centre	Other	Store °	At Work
Boys & Girls, 2–3 year	1731(40)	27(7)	76(14)	0(.)	5.7(1.5)	0.2(0.1)	0.8(0.4)	1.4(1.0)	4.2(2.0)	80.2(11.3)	2.3(1.1)	123.8(21.5)	3.0(1.8)	4.0(1.7)	0(0)
Boys & Girls, 4–8 year	2270(41)	35(6)	115(14)	0(0)	31.9(5.8)	0.9(0.3)	4.8(2.1)	4.4(1.7)	37.3(6.6)	38.1(7.5)	8.2(5.4)	111.7(11.5)	7.0(1.6)	12.3(4.3)	0.1(0.1)
Boys, 9–13 year	2876(57)	145(33)	175(16)	3.3(2.3)	62.8(11.9)	1.8(0.6)	4.6(2.7)	15.5(5.0)	61.7(16.3)	7.3(4.0)	2.5(1.6)	96.5(12.9)	13.0(5.0)	17.0(3.8)	0(0)
Girls, 9–13 year	2498(62)	78(17)	125(12)	0.1(0.1)	33.3(5.8)	1.3(0.4)	10.0(5.2)	11.4(4.2)	47.5(7.6)	3.9(2.0)	0.4(0.2)	125.5(15.9)	11.9(4.1)	12.8(4.2)	0(0)
Boys, 14–18 year	3196(75)	144(18)	401(29)	5.0(2.1)	101.5(16.0)	18.3(8.1)	6.2(2.0)	17.8(4.5)	85.5(12.1)	0(0)	0.6(0.4)	101.6(13.7)	19.5(8.3)	24.9(4.7)	3.4(1.9)
Girls, 14–18 year	2171(52)	116(16)	294(25)	1.1(0.5)	60.8(8.9)	4.5(1.1)	13.2(4.4)	19.4(4.8)	98.2(12.6)	0.1(0.1)	2.5(1.6)	102.8(13.3)	18.2(4.7)	25.8(7.1)	4.1(1.9)
Men, 19–30 year	2896(108)	287(31)	438(35)	37.6(11.5)	122.4(20.5)	18.4(7.9)	11.8(3.3)	25.3(6.1)	43.5(24.2)	0.2(0.2)	0.4(0.3)	104.7(20.7)	9.5(3.8)	56.5(15.7)	11.6(3.2)
Women, 19–30 year	2000(71)	187(21)	245(21)	7.8(2.7)	110.5(21.1)	5.2(1.5)	9.9(4.7)	13.5(2.8)	21.9(6.4)	1.7(1.0)	4.2(1.5)	89.3(17.6)	10.8(4.3)	17.6(3.7)	13.0(3.6)
Men, 31–50 year	2781(70)	202(22)	274(26)	14.8(3.3)	76.9(12.6)	27.4(13.4)	9.5(5.1)	50.8(9.5)	2.5(1.2)	0(0)	0.7(0.4)	94.2(25.1)	23.5(11.3)	30.8(7.7)	19.4(4.4)
Women, 31–50 year	2140(55)	227(27)	150(16)	5.1(1.8)	59.8(9.2)	4.6(1.5)	14.4(4.1)	52.8(11.4)	11.4(5.3)	1.7(1.4)	3.1(1.8)	63.7(12.8)	5.7(2.3)	16.6(2.7)	15.9(3.3)
Men, 51–70 year	2772(58)	270(34)	115(14)	7.4(2.3)	43.0(9.4)	3.2(1.1)	15.3(7.0)	25.2(5.7)	0.8(0.6)	0(0)	1.0(0.4)	38.9(7.2)	12.5(4.5)	21.3(8.0)	7.4(1.6)
Women, 51–70 year	2140(47)	162(17)	91(13)	9.6(4.4)	23.1(3.9)	1.3(0.3)	7.8(2.4)	24.4(5.1)	0.7(0.3)	0(0)	6.6(2.5)	70.8(9.5)	15.8(7.0)	10.9(2.4)	6.9(1.9)
Men, >70 year	2483(65)	165(32)	75(17)	4.4(2.1)	14.8(4.3)	0.4(0.4)	1.0(0.8)	10.6(3.3)	0(.)	0(0)	48.0(29.2)	42.4(8.9)	15.0(5.5)	4.5(1.9)	0.1(0.1)
Women, >70 year	2033(54)	106(14)	37(6)	1.0(0.5)	11.1(4.2)	0.3(0.2)	4.6(2.2)	17.2(4.3)	0.8(0.7)	0(0)	26.8(7.3)	50.2(8.0)	8.9(4.5)	3.1(1.1)	0.1(0)

Data are presented as Mean (Standard Error of the Mean).
